# RIPK1 and RIPK3 are positive prognosticators for cervical cancer patients and C2 ceramide can inhibit tumor cell proliferation *in vitro*

**DOI:** 10.3389/fonc.2023.1110939

**Published:** 2023-05-01

**Authors:** Tilman L. R. Vogelsang, Verena Kast, Konstantin Bagnjuk, Katja Eubler, Sree Priyanka Jeevanandan, Elisa Schmoeckel, Anna Trebo, Nicole Elisabeth Topalov, Sven Mahner, Doris Mayr, Artur Mayerhofer, Udo Jeschke, Aurelia Vattai

**Affiliations:** ^1^ Department of Obstetrics and Gynecology, University Hospital, Ludwig-Maximilians-University (LMU) Munich, Munich, Germany; ^2^ Biomedical Center Munich (BMC), Cell Biology, Anatomy III, Ludwig-Maximilians-University (LMU) Munich, Planegg, Germany; ^3^ Faculty of Medicine, Institute of Pathology, Ludwig-Maximilians-University (LMU) Munich, Munich, Germany; ^4^ Department of Obstetrics and Gynecology, University Hospital Augsburg, Augsburg, Germany

**Keywords:** necroptosis, programmed cell death, cervical cancer, human papillomavirus, RIPK1, RIPK3, pMLKL, C2 ceramide

## Abstract

**Introduction:**

The enzymes Receptor-interacting serine/threonine-protein kinase 1 (RIPK1) und 3 (RIPK3) as well as the protein Mixed lineage kinase domain like pseudokinase (pMLKL) play a role in the signaling cascade of necroptosis. This is a form of programmed cell death which is caspase-independent. High-risk human papilloma virus infection can inhibit necroptosis. Thereby, a persistent infection and consequently the development of cervical cancer can be triggered. Aim of this study was the analysis of the expression of RIPK1, RIPK3 and pMLKL in cervical cancer tissue and the evaluation of its prognostic value on overall survival, progression-free survival and additional clinical parameters.

**Methods:**

The expression of RIPK1, RIPK3, and pMLKL in cervical cancer tissue microarrays of n = 250 patients was analyzed immunohistochemically. Further, the effect of C2 ceramide on several cervical cancer cell lines (CaSki, HeLa, SiHa) was examined. C2 ceramide is a biologically active short-chain ceramide that induces necroptosis in human luteal granulosa cells.

**Results:**

Significantly longer overall survival and progression-free survival rates could be detected in cervical cancer patients expressing nuclear RIPK1 or RIPK3 alone or simultaneously (RIPK1 and RIPK3). Cell viability and proliferation was reduced through C2 ceramide stimulation of cervical cancer cells. Simultaneous stimulation of C2 ceramide and the pan-caspase inhibitor Z-VAD-fmk, or the RIPK1-inhibitor necrostatin-1, partly reversed the negative effect of C2 ceramide on cell viability. This observation could imply that caspase-dependent and -independent forms of cell death, including necroptosis, can occur. AnnexinV-FITC apoptosis staining induced a significant increase in apoptotic cells in CaSki and SiHa cells. The stimulation of CaSki cells with C2 ceramide led to a significant percentual increase in necrotic/intermediate (dying) cells after stimulation with C2 ceramide. In addition, after stimulation with C2 ceramide, CaSki and HeLa cells live cell imaging showed morphological changes which are common for necroptosis.

**Discussion:**

In conclusion, RIPK1 and RIPK3 are independent positive predictors for overall survival and progression-free survival in cervical cancer patients. C2 ceramide can reduce cell viability and proliferation in cervical cancer cells by inducing most likely both apoptosis and necroptosis.

## 1 Introduction

Necroptosis is a caspase-independent form of regulated lytic cell death which was first described in 2005 as a contributor to delayed ischemic brain injury *in vivo* (1). Even though it is morphologically similar to necrosis (organelle swelling, plasma membrane rupture, inflammatory response), necroptosis is dependent on a highly regulated signaling cascade (2). Necroptosis might have evolved as a host defense mechanism against intracellular pathogens encoding caspase 8 (casp8) inhibitors (3, 4) but is of high pathophysiological relevance in several medical conditions such as cardiovascular diseases (atherosclerosis (5), myocardial infarction and stroke (6–8), ischemia-reperfusion injury (9)), pancreatitis (10), inflammatory bowel disease and necrotizing enterocolitis (4, 11–13). In cancer, necroptosis is of interest since escape of regulated cell death and inflammation play an important role in tumorigenesis (14).

Cervical cancer is the fourth most common cancer in females worldwide with approximately 604.000 new cases and an estimated 342.000 deaths in 2020 (15). Over the last 30 years the high incidence rate could be reduced due to early diagnosis and effective prevention, screening (i.e. Pap smear test) and treatment programs (16, 17). Persistent infection with high-risk human papilloma virus (hr-HPV) is a key factor for cervical cancer to develop (18). The viral oncoproteins high-risk E6 (hr-E6) and high-risk E7 (hr-E7) disturb the cell cycle, promote hyperproliferation and genomic instability and block apoptosis to promote immortalization (19). Furthermore, hr-HPV suppresses the induction of necroptosis by downregulating the expression of its key regulator receptor-interacting serine/threonine-protein kinase 3 (RIPK3) allowing infected keratinocytes to partially evade the immune system and potentially lead to the progression of hr-HPV induced lesions (20).

Necroptosis is induced *via* activation of Z-DNA binding protein 1 (ZBP1, also known as DAI and DLM-1), tumor necrosis factor receptor 1 (TNFR1), toll-like receptor 3 (TLR3)-TIR domain-containing adaptor protein inducing IFNβ (TRIF), TLR4-TRIF (21) or interferons (22). The necroptotic pathway critically depends on MLKL and RIPK3 (23). After activation, e.g., through binding of ZBP1 to RIPK3 (21), RIPK3 oligomerizes and forms a complex with mixed lineage kinase domain-like protein (MLKL) leading to its phosphorylation (pMLKL) which induces membrane pore induction, disruption of membrane integrity and the release of cell DAMPs (24–26). The role of Receptor-interacting serine/threonine-protein kinase 1 (RIPK1) in necroptosis is more complex, since RIPK1 plays an important role in several pathways. Ligation of TNFR1 can lead to either induction of the NF-κB pathway (gene expression of proinflammatory and prosurvival factors), apoptosis, or necroptosis (27–30). After ligation of TNFR1, RIPK1 among others is recruited and ubiquitinated mediated by cellular inhibitor of apoptosis protein 1 and 2 (cIAP1/2) leading to the induction of the NF-κB pathway (31). When RIPK1 is deubiquitylated by the absence or inhibition of cIAP1/2, RIPK1 is released from TNFR1 and recruits Fas-ligand associated protein with death domain (FADD) which itself recruits procaspase 8 (procasp8) (32). Procasp8 then oligomerizes and autocatalyzes to autoactivate and induce apoptosis (32). When casp8 is inhibited, RIPK3 is activated through autophosphorylation leading to the induction of necroptosis (32).

Ceramide is a central molecule in sphingolipid metabolism which can function as a tumor suppressor lipid (33), inducing apoptotic responses in neuroblastoma cells (34) and breast cancer cells (35). In human luteal granulosa cells necroptosis can be induced through stimulation with the soluble C2 ceramide (36).

Aim of this study was to analyze the expression of RIPK1, RIPK3, and pMLKL in cervical carcinoma patient tissue and to correlate their expression with clinical parameters, overall survival (OS) and progression-free survival (PFS). Another goal was the stimulation of cervical carcinoma cell lines CaSki, HeLa, and SiHa with C2 ceramide and to characterize its impact on induction of necroptosis or apoptosis, cell viability and cell proliferation.

## 2 Materials and methods

### 2.1 Characteristics of patients and biopsies

All assessable cervical cancer patients (*n* = 250) who had undergone surgery for treatment of cervical cancer at the Department of Gynecology and Obstetrics, Ludwig-Maximilians-University Munich, Germany, from 1993 until 2002 and whose paraffin-embedded tumor tissue was available were included in this study. Patients were not pre-selected. An experienced gynecological pathologist assigned histopathological tumor subtypes (squamous cell carcinoma, adenocarcinoma, adenosquamous carcinoma) and grading (G1: well differentiated, G2: moderately differentiated, G3: poorly differentiated). TNM classification (T = primary tumor site, N = regional lymph node involvement, M = presence of distant metastatic spread) was carried out as published by the Union for International Cancer Control (UICC). FIGO classification was evaluated according to the criteria of the Fédération Internationale de Gynécologie et d’Obstétrique (FIGO). Clinical and patient follow-up data was received from the Munich Cancer Registry (**Table 1**). The mean age of patients at diagnosis was 49.0 ± 13.0 years with a range between 20.4 and 83.3 years. Patients who died independently of their tumor or whose cause of death is unknown were censured. Other histopathological parameters were examined and published previously, enabling correlation analysis to the parameters investigated in this study.

**Table 1:** Description of clinical and pathological variables of the patients.

	number of cases (total number of cases: *n*=250)	%
histopathological tumor subtype		
squamous cell carcinoma	194	77.6
adenocarcinoma	34	13.6
adenosquamous carcinoma	15	6.0
unknown	7	2.8
tumor grading		
G1	21	8.4
G2	143	57.2
G3	78	31.2
unknown	8	3.2
extent of primary tumor (pT)		
pT1	107	42.8
pT2	126	50.4
pT3	9	3.6
pT4	1	0.4
unknown	7	2.8
regional lymph node involvement (pN)		
pN0	145	58.0
pN1	98	39.2
unknown	7	2.8
presence of distant metastatic spread (pM)		
pM0	2	0.8
pM1	7	2.8
pMX	235	94
unknown	6	2.4
FIGO classification		
FIGO I	79	31.6
FIGO II	64	25.6
FIGO III	93	37.2
FIGO IV	7	2.8
unknown	7	2.8
progression		
none	180	72.0
at least one	63	25.2
unknown	7	2.8
survival		
censured	211	84.4
dead (tumor-dependent)	33	13.2
unknown	6	2.4

### 2.2 Immunohistochemistry

Directly after surgical resection, the samples were fixed in neutral-buffered formalin (3.7%) followed by standardized paraffin embedding. Tissue micro arrays (TMAs) were then prepared from formalin fixed paraffin embedded (FFPE) cervical cancer samples. The tissue sections (3µm) were dewaxed in Roticlear (Carl Roth GmbH + Co. KG, Karlsruhe, Germany) for 20 min followed by immersion in 3% H_2_O_2_ in methanol at room temperature (RT) for 20 min to inactivate endogenous peroxidase. A descending ethanol gradient (S99%, 70% and 50%) was used to rehydrate the specimens. The sections were prepared for epitope retrieval in a pressure cooker for 5 min using sodium citrate buffer (pH 6.0; 0.1 mol/L citric acid/0.1 mol/L sodium citrate) followed by washing in distilled water and phosphate buffer saline (PBS) (RIPK1 and RIPK3 staining) or Tris buffered saline (TBS) (pMLKL staining). To block non-specific binding of primary antibodies, Blocking Solution (reagent 1, ZytoChem Plus HRP Polymer System (Mouse/Rabbit); Zytomed Systems GmbH, Berlin, Germany) was applied for 5min at RT for RIPK1 and RIPK3 staining. For pMLKL staining, non-specific binding of primary antibodies was blocked by applying blocking serum (= two drops of normal goat serum diluted in 10ml sterile TBS; VECTASTAIN Elite ABC-HRP Kit, Peroxidase (Rabbit IgG) – PK6101; Vector Laboratories, Inc., Burlingame, CA, USA) for 20 min at RT. Specimens were then separately incubated with the following primary antibodies: anti-RIPK1 (polyclonal rabbit IgG, HPA015257; Sigma Aldrich, St. Louis, MO, USA) for 17h at 4°C followed by 30min at RT in a 1:50 dilution in PBS, anti-RIPK3 (polyclonal rabbit IgG;, HPA055087 Sigma Aldrich, St. Louis, MO, USA) for 16h at 4°C in a 1:1500 dilution in PBS and anti-MLKL (phospho S358) (monoclonal Rabbit IgG, ab187091; Abcam, Cambridge, UK) for 16h at 4°C in a 1:100 dilution in TBS. Then, the tissue slides for RIPK1 and RIPK3 staining were incubated with PostBlock Reagent (reagent 2) for 20 min and HRP-Polymer (Mouse/Rabbit) (reagent 3) for 30 min which contain secondary antibodies (anti-mouse/-rabbit) and peroxidase according to the manufacturer’s protocol (reagent 2, reagent 3, ZytoChem Plus HRP Polymer System (Mouse/Rabbit); Zytomed Systems GmbH, Berlin, Germany). The tissue slides for pMLKL staining were incubated with the secondary antibody (= two drops of normal goat serum and one drop of biotinylated goat anti-rabbit IgG diluted in 10ml sterile TBS; VECTASTAIN Elite ABC-HRP Kit, Peroxidase (Rabbit IgG) – PK6101) for 30 min followed by incubation with ABC complex (= four drops of Reagent A and four drops of Reagent B diluted in 10ml sterile TBS; VECTASTAIN Elite ABC-HRP Kit, Peroxidase (Rabbit IgG) – PK6101) for 30 min. All slides were washed with PBS (RIPK1, RIPK3) or TBS (pMLKL) after every incubation step. Next, 3,3′-diaminobenzidine chromogen (DAB; Dako, Glostrup, Denmark) and the according substrate buffer (Liquid DAB and Substrate Chromogen System, DAKO, Munich, Germany) were added to the slides. The slides were washed in distilled water to stop the reaction followed by counterstaining in Mayer’s acidic hematoxylin. The slides were then immersed in an ascending ethanol gradient and Roticlear and finally cover slipped using ROTI^®^ Mount (Carl Roth GmbH + Co. KG, Karlsruhe, Germany). Appropriate tissue was used as positive control (placenta for RIPK1, RIPK3 and pMLKL) and negative controls (placenta for RIPK1 and pMLKL and colon for RIPK3) (**Figure S2**). For the negative controls the primary antibodies were each replaced with a specific isotype control antibody (BioGenex, Fremont, CA, USA).

### 2.3 Quantification

The immunohistochemically stained cervical cancer specimens were examined using a Leitz Diaplan photomicroscope (Leitz, Wetzlar, Germany). Quantification of RIPK1, RIPK3, and pMLKL expression was performed by applying the semiquantitative immunoreactive score (IRS) which evaluates the intensity and distribution pattern of antigen expression (37). The IRS is calculated by multiplying the number of positively stained cells (in %) (0: no staining; 1: 1% - 10% stained tumor cells; 2: 11% - 50% stained tumor cells; 3: 51% - 80% stained tumor cells; 4: > 80% stained tumor cells) with the predominant staining intensity (0: none; 1: weak; 2: moderate; 3: strong). The scale goes from 0 (no expression) to 12 (very high expression). Images were taken with Flexacam C1 (Leica Microsystems (Switzerland) Ltd., Heerbrugg, Switzerland).

### 2.4 Cell lines

The human cervical cancer cell lines CaSki (epidermoid carcinoma), HeLa (adenocarcinoma) and SiHa (squamous cell carcinoma) obtained from ATCC (Rockville, MD, USA) were used for *in vitro* studies. They were maintained in culture using RPMI Medium 1640 (1X) + GlutaMAX (ThermoFisher Scientific, Waltham, MA, USA) supplemented with 10% fetal bovine serum at standard conditions (humidified incubator at 37°C and 5% CO_2_ saturation).

### 2.5 MTT assay

Cell viability was analyzed using Thiazolyl Blue Tetrazolium Bromide (MTT; Sigma Aldrich, St. Louis, MO, USA; M5655). After 24h, 48h, and 72h of stimulation incubation, 20µl of Thiazolyl Blue Tetrazolium Bromide (5mg MTT/ml sterile PBS) was added to each well followed by soft shaking on a plate shaker for 5 min and incubation for 1.5h at 37°C in a 5% CO_2_ humidified atmosphere. Then, the supernatant was removed and 200µl dimethyl sulfoxide (DMSO)/well was added to solubilize the blue crystals. After 5 min of soft shaking on a plate shaker, optical density was evaluated at wavelengths of 595nm by the Elx800 universal microplate reader (BioTek Instruments GmbH, Bad Friedrichshall, Germany) and Gen5 software (BioTek Instruments GmbH, Bad Friedrichshall, Germany). All tests were repeated three times with 5 wells per concentration.

### 2.6 BrdU assay

For analysis of cell proliferation, the colorimetric bromodeoxyuridine (BrdU) assay was performed using the Cell Proliferation ELISA, BrdU (colorimetric) kit from Roche (F. Hoffmann-La Roche AG, Basel, Switzerland). The assay was performed according to the manufacturer’s protocol. CaSki, HeLa and SiHa cells were incubated with C2 ceramide for 24h, 48h and 72h in 96-well plates as described below. Then, BrdU was added, and the cells were incubated for another 24h. The medium was removed and 200µl FixDenat per well was added to fix the cells and denature the DNA for improvement of accessibility of the incorporated BrdU for detection by the antibody. Next, anti-BrdU-POD was added and bound to the BrdU incorporated in newly synthesized DNA followed by incubation for 90 min at RT. The supernatant was removed, and the wells were washed three times with a washing solution. 100µl substrate solution (tetramethyl-benzidine, TMB) per well was added and incubated in the dark at RT to detect the immune complexes. Visualization of the substrate reaction was an increasing deep blue coloration. After 15 min of incubation, the reaction was stopped by adding sulfuric acid. The plate was then measured at a wavelength of 450nm using the Elx800 universal microplate reader using Gen5 software. All tests were repeated three times and with 5 wells per concentration.

### 2.7 Stimulation with C2 ceramide

For MTT- and BrdU assay, 5x10^3^ CaSki, HeLa and SiHa cells were incubated in 100µl RPMI Medium 1640 (1X) + GlutaMAX (ThermoFisher Scientific, Waltham, MA, USA) supplemented with 10% FBS in 96-well plates (flat bottom) for four hours. Then, medium with 10% FBS was removed and 100µl medium without FBS was added followed by 24h of incubation.

Stimulation with 50µM, 100µM or 200µM C2 ceramide (Enzo Life Sciences (ELS) AG, Lausen, Switzerland; BML-SL100, Prod.Nr.: ALX-306-024) was performed by dissolving the substance to 200mM (68.3mg/ml) in DMSO followed by dilution of the ceramide/DMSO stock 200-fold into a BSA solution which was prepared using fatty acid-free BSA diluted in water to 66mg/ml (1mM). At first, a precipitate was formed but dissolved after 30-60 min of stirring at RT. Then, the ceramide/DMSO/BSA solution was diluted in 100µl medium without FBS at respective concentrations (100µM, 200µM or 400µM) and added to the wells. The 1:1 dilution in the well gave the final concentration of 50µM, 100µM or 200µM.

Necrostatin-1 (nec-1, Santa Cruz Biotechnology, Inc., Heidelberg, Germany; CAS 4311-88-0) and Z-VAD-fmk (Selleck Chemicals, Munich, Germany; Catalog No. S7023, CAS 187389-52-2) were both dissolved to 50mM and further to 5mM in DMSO. Co-stimulation of C2 ceramide and nec-1 or C2 ceramide and Z-VAD-fmk was initiated by pre-stimulation with 1µM, 5µM, 20µM or 50µM nec-1 or 1µM, 5µM, 20µM or 50µM Z-VAD-fmk for 2h by diluting the substances in 100µl medium without FBS at respective concentrations (2µM, 10µM, 40µM or 100µM) and adding it to the wells. The 1:1 dilution in the well gave the final concentration of 1µM, 5µM, 20µM or 50µM. Then, the supernatant was removed and co-stimulation of 100µM C2 ceramide and 1µM, 5µM, 20µM or 50µM nec-1 or 100µM C2 ceramide and 1µM, 5µM, 20µM or 50µM Z-VAD-fmk was performed by diluting the substances in 200µl medium without FBS at respective concentrations (100µM C2 ceramide and 1µM, 5µM, 20µM or 50µM nec-1/Z-VAD-fmk) and adding it to the wells. The concentrations of the substances were based on previous studies (36, 38, 39). Unstimulated cells and wells without any cells served as negative controls. A solvent control (DMSO) was always conducted.

### 2.8 Live-cell imaging

For visualization of C2 ceramide’s effect on cervical carcinoma cells, live-cell imaging was performed. Therefore, 5 x10^4^ CaSki, HeLa, and SiHa cells were seeded in a glass-bottomed culture dish (µ-Dish, Ø 35 mm; ibidi, Gräfelfing, Germany) and incubated under standard conditions at 37°C and 5% CO_2_. The cells were then serum starved for 6h before being stimulated with C2 ceramide by dissolving C2 ceramide as described above. 100 µM of C2 ceramide diluted in medium without FBS was added to the cells, and cells were imaged for 72 h using an Axiovert 135 microscope (Carl Zeiss). To produce a time-lapse series (Micro-Manager 1.3 Microscopy Software Ron Vale’s laboratory at UCSF, USA), images (ProgRes MF, Jenoptik, Jena, Germany) were taken every 20 minutes. iMovie 9.0.3 (Apple Inc., Cupertino, CA, USA) was used to create movie sequences.

### 2.9 Western blot

Cells were cultured under basal conditions and harvested. The method was described before (36). In brief, cellular protein was isolated using RIPA buffer containing protease and phosphatase inhibitors (PI, Thermo Fisher Scientific, Waltham, USA) and 16 µg were loaded onto a SDS-PAGE gel. Primary antibody (anti-RIPK3, polyclonal rabbit IgG, HPA055087, Lot: R73206, Sigma Aldrich, St. Louis, MO, USA) and secondary antibody (IRDye^®^ 680RD Donkey anti-Rabbit IgG, Secondary Antibody) were used.

### 2.10 Cell death detection by flow cytometry

For quantitative detection of cell death, Annexin V-FITC (ALX-209-256-T100, Enzo Life Sciences, Farmingdale, NY, USA) and SYTOX™ Red Dead Cell Stain (S34859, Invitrogen, Carlsbad, CA, USA) based flow cytometry was performed. Briefly, 2 x 10^5^ CaSki, HeLa, or SiHa cells were seeded and after a 6h starvation step on medium without FBS the next day cells were treated with 100µM C2 ceramide or the solvent control for 72h, as described above. Cells were trypsinized, washed in PBS and resuspended in a buffer consisting of 10mM HEPES, 140mM NaCl and 2.5mM CaCl_2_ at pH 7.4, followed by incubation with Annexin V-FITC for 10 minutes according to the manufacturer’s instructions, and addition of SYTOX Red Dead Cell Stain (1:1000). Labelled cells were sorted using the BD FACSCanto™ II (Becton, Dickinson and Company, Franklin Lakes, NJ, USA) and signals (Annexin V-FITC: 488nm, 530/30 bandpass filter; SYTOX™ Red Dead Cell Stain: 633nm excitation, 660/20 bandpass filter) were analyzed with the BD FACSDiva Software (version 8.0.1, Becton, Dickinson and Company). This was repeated two times (*n* = 2).

### 2.11 Statistical analysis

For statistical analysis, IBM SPSS Statistics Version 26.0.0.0 (IBM, Armonk, New York, NY, USA) was used. *p*-values of *p* < 0.05 were considered statistically significant. Kruskal-Wallis-H test was applied as appropriate for group comparisons of independent groups regarding clinical and pathological subgroups. Bivariate correlations between staining results in this study and other variables and clinicopathological data were determined using Spearman’s rank correlation coefficient. Differences in OS and PFS times of cervical cancer patients were tested for significance by log-rank (Mantel-Cox) test and visualized using Kaplan-Meier curves. Cox regression analysis was used to ascertain the independence of the investigated prognosticators. Variables included in the Cox regression model were patient’s age, histological subtype, tumor grading, FIGO, nodal status, RIPK1, RIPK3, co-expression of RIPK1 and RIPK3, and co-expression of RIPK1, RIPK3, and pMLKL. RIPK1, RIPK3, and pMLKL expression were divided into low and high expression for survival analysis. All *in vitro* analyses were statistically analyzed using Wilcoxon test and visualized with GraphPad Prism 7.00 (San Diego, CA, USA). For statistical analysis of AnnexinV-FITC apoptosis staining followed by FACS analysis the percentual number of apoptotic cells after treatment was compared to the percentual number of apoptotic cells in the control and the percentual number of necrotic and intermediate (dying) cells after treatment was compared to the percentual number of necrotic and intermediate (dying) cells in the control using Wilcoxon test.

### 2.12 Ethical approval and informed consent

The study was approved by the local ethics committee of Ludwig-Maximilians University of Munich, Germany (approval number 259-16, 2016). All tissue samples examined in this study were obtained from material from the archives of the Department of Gynecology and Obstetrics, Ludwig-Maximilians University of Munich. The material used in this study was fully anonymized, declared as left over after finalizing all diagnostic procedures and was used more than 10 years after surgery. All experiments involving human participants were performed according to the standards of the Declaration of Helsinki from 1964 and its later amendments and were in accordance with the ethical standards of the institutional and/or national research committee. During analyses, the observers were fully blinded for patients’ data.

## 3 Results

### 3.1 Expression of RIPK1, RIPK3, and pMLKL in cervical cancer and correlation with various clinical and pathological parameters

Immunohistochemical staining showed that RIPK1 and pMLKL were expressed in the nucleus and cytoplasm while RIPK3 was expressed in the nucleus in cervical cancer tissue. Differences in expression of RIPK1, RIPK3, and pMLKL were examined by comparing the immunoreactive score (IRS) in the groups of histological subtypes (squamous cell carcinoma, adenocarcinoma, adenosquamous carcinoma), grading (G1-G3), TNM- and FIGO-classification.

#### 3.1.1 Correlation of RIPK1, RIPK3, and pMLKL with grading and histology

**Figure 1:**
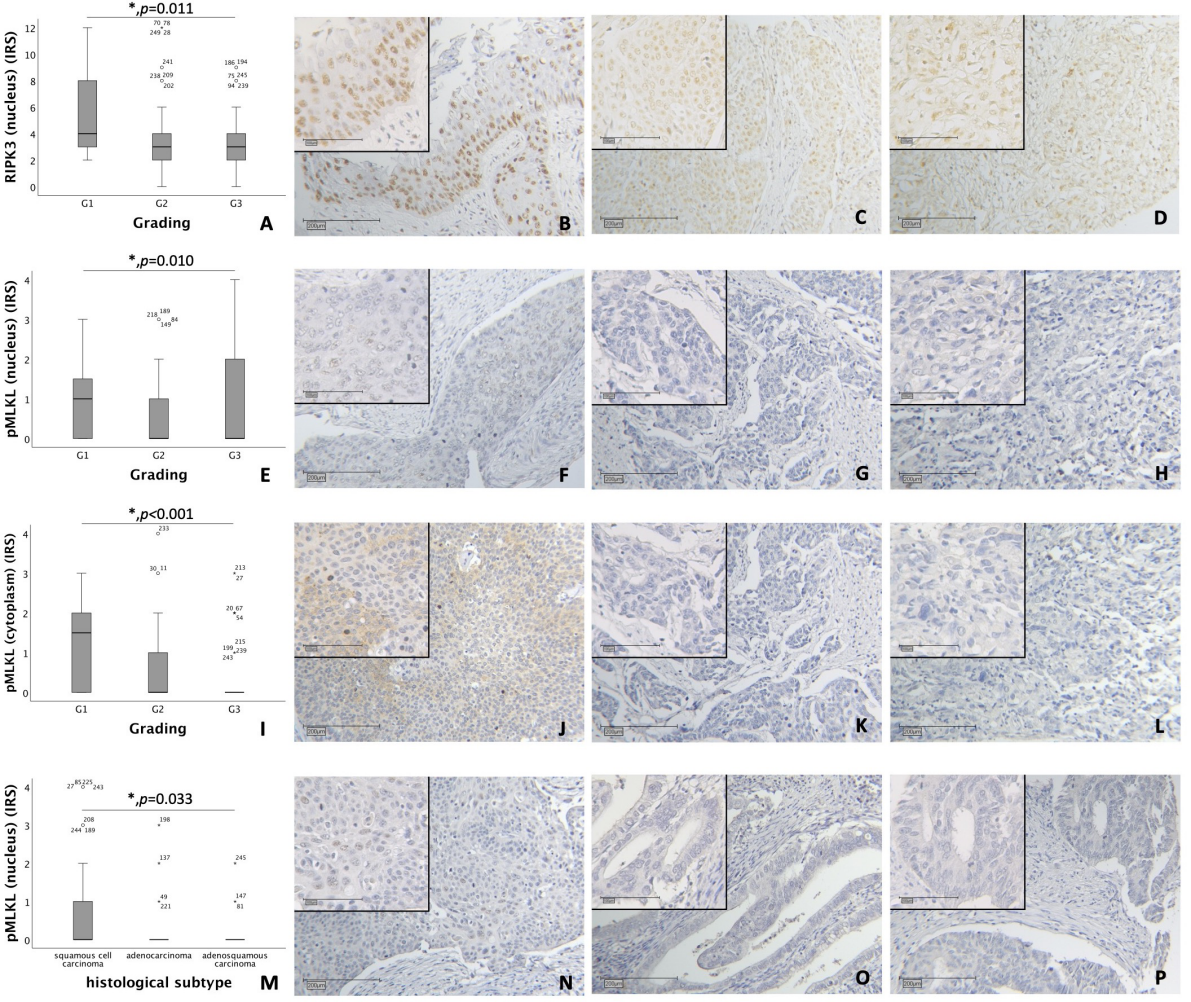
**(A–D)** Correlation of nuclear RIPK3 expression with tumor grading in cervical carcinoma (p = 0.011). **(A)** boxplot of nuclear RIPK3 expression and tumor grading in cervical carcinoma. **(B)** cervical carcinoma, grading G1 (n = 18) with a nuclear RIPK3 IRS of 6, magnification x10 and x25 in the inset. **(C)** cervical carcinoma, grading G2 (n = 131) with a nuclear RIPK3 IRS of 3, magnification x10 and x25 in the inset. **(D)** cervical carcinoma, grading G3 (n = 74) with a nuclear RIPK3 IRS of 3, magnification x10 and x25 in the inset. **(E–H)** Correlation of nuclear pMLKL expression with tumor grading in cervical carcinoma (p = 0.010). **(E)** boxplot of nuclear pMLKL expression and tumor grading in cervical carcinoma. **(F)** cervical carcinoma, grading G1 (n = 16) with a nuclear pMLKL IRS of 3, magnification x10 and x25 in the inset. **(G)** cervical carcinoma, grading G2 (n = 119) with a nuclear pMLKL IRS of 0, magnification x10 and x25 in the inset. **(H)** cervical carcinoma, grading G3 (n = 72) with a nuclear pMLKL IRS of 0, magnification x10 and x25 in the inset. **(I–L)** Correlation of cytoplasmic pMLKL expression with tumor grading in cervical carcinoma (p < 0.001). **(I)** boxplot of cytoplasmic pMLKL expression and tumor grading in cervical carcinoma. **(J)** cervical carcinoma, grading G1 (n = 16) with a cytoplasmic pMLKL IRS of 3, magnification x10 and x25 in the inset. **(K)** cervical carcinoma, grading G2 (n = 119) with a cytoplasmic pMLKL IRS of 0, magnification x10 and x25 in the inset. **(L)** cervical carcinoma, grading G3 (n = 72) with a cytoplasmic pMLKL IRS of 0, magnification x10 and x25 in the inset. **(M–P)** Correlation of nuclear pMLKL expression with histological subtype in cervical carcinoma (p = 0.033). **(M)** boxplot of nuclear pMLKL expression and histological subtype in cervical carcinoma. **(N)** cervical squamous cell carcinoma (n = 169) with a nuclear pMLKL IRS of 2, magnification x10 and x25 in the inset. **(O)** cervical adenocarcinoma (n = 23) with a nuclear pMLKL IRS of 0, magnification x10 and x25 in the inset. **(P)** cervical adenosquamous carcinoma (n = 13) with a nuclear pMLKL IRS of 0, magnification x10 and x25 in the inset.

Nuclear RIPK3 expression was significantly higher in cervical cancer tissue with low grading (G1) compared to grading G2 and G3 (*p* = 0.011) (**Figures 1A–D**).

Nuclear pMLKL expression correlated significantly with low grading (G1) in comparison to grading G2 and G3 (*p* = 0.010) (**Figures 1E–H**). In grading G1, cytoplasmic pMLKL was expressed significantly higher compared to grading G2 and G3 (*p* < 0.001) (**Figures 1I–L**).

A comparison of grading with nuclear and cytoplasmic RIPK1 using Kruskal-Wallis test showed no significant correlation.

Differences in nuclear pMLKL expression regarding histological subtypes (squamous cell carcinoma, adenocarcinoma, adenosquamous carcinoma) were significant (*p* = 0.033) with similar medians (IRS = 0) (**Figures 1M–P**). No significant correlation was found when comparing histological subtype with nuclear or cytoplasmic RIPK1 expression, nuclear RIPK3 expression or cytoplasmic pMLKL expression.

#### 3.1.2 Correlation of RIPK1, RIPK3, and pMLKL with TNM- and FIGO-classification

**Figure 2:**
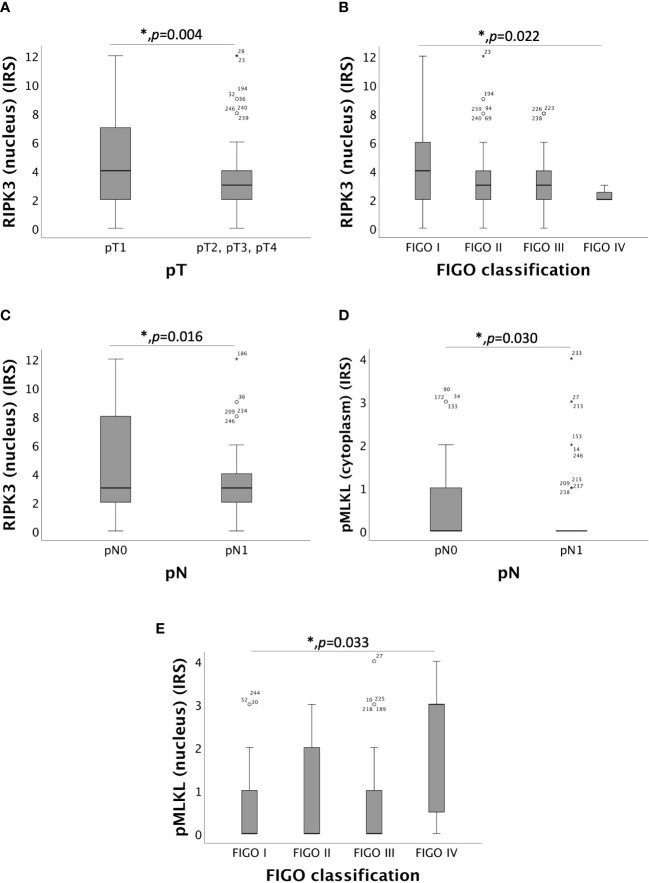
Correlation of RIPK3 and pMLKL with pT, pN and FIGO-classification. **(A)** boxplot of nuclear RIPK3 expression and the size or the extent of the primary tumor (pT) in cervical carcinoma (p = 0.004), median IRS of nuclear RIPK3 in pT1 was 4 (n = 96) and in pT2, pT3 and pT4 was 3 (n = 130). **(B)** boxplot of nuclear RIPK3 expression and FIGO classification in cervical carcinoma (p = 0.022), median IRS of nuclear RIPK3 in FIGO I was 4 (n = 69), in FIGO II was 3 (n = 61), in FIGO III was 3 (n = 89) and in FIGO IV was 2 (n = 7). **(C)** boxplot of nuclear RIPK3 expression and regional lymph node status (pN) in cervical carcinoma (p = 0.016), median IRS of nuclear RIPK3 expression in pN0 was 3 (n = 133) and in pN1 was 3 (n = 93). **(D)** boxplot of cytoplasmic pMLKL expression and regional lymph node status (pN) in cervical carcinoma (p = 0.030), median IRS of cytoplasmic pMLKL expression in pN0 was 0 (n = 120) and in pN1 was 0 (n = 87). **(E)** boxplot of nuclear pMLKL expression and FIGO classification in cervical carcinoma (p = 0.033), median IRS of nuclear pMLKL in FIGO I was 0 (n = 61), in FIGO II was 0 (n = 58), in FIGO III was 0 (n = 81) and in FIGO IV was 3 (n = 7).

Nuclear RIPK3 overexpression significantly correlated with low pT (pT1) with a median IRS of 4 in comparison to pT2, pT3 and pT4 with a median IRS of 3 (*p* = 0.004) (**Figure 2A**). Increasing grade of FIGO was associated with decreasing expression of RIPK3 in the nucleus (*p* = 0.022) (**Figure 2B**). Differences in the expression of nuclear RIPK3 regarding regional lymph node involvement (pN) were significant (*p* = 0.016) with similar medians (IRS = 3) (**Figure 2C**). Expression of cytoplasmic pMLKL correlated with pN showed significant (*p* = 0.030) differences with similar medians (IRS = 0) (**Figure 2D**). A small subgroup of patients diagnosed with cervical carcinoma FIGO IV (*n* = 7) had a significantly (*p* = 0.033) higher nuclear pMLKL expression (IRS = 3) (**Figure 2E**).

The correlation between nuclear and cytoplasmic RIPK1 expression and TNM- and FIGO classification showed no significance.

#### 3.1.3 Correlation of RIPK1, RIPK3, and pMLKL with histopathological parameters

Spearman’s rank correlation coefficient was evaluated for RIPK1, RIPK3, pMLKL and various histopathological markers which were stained and analyzed in the applied cervical cancer patient collective of previous studies (**Tables S1, S2, S3**). RIPK1, RIPK3, and pMLKL showed significant correlations with each other. Nuclear RIPK1 significantly correlated with cytoplasmic RIPK1 (Spearman rho: 0.486, *p* < 0.001), nuclear RIPK3 (Spearman rho: 0.414, *p* < 0.001), nuclear pMLKL (Spearman rho: 0.184, *p* = 0.009) and cytoplasmic pMLKL (Spearman rho: 0.310, *p* < 0.001). Cytoplasmic RIPK1 significantly correlated with RIPK3 (Spearman rho: 0.471, *p* < 0.001), nuclear pMLKL (Spearman rho: 0.168, *p* = 0.016) and cytoplasmic pMLKL (Spearman rho: 0.278, *p* < 0.001). Nuclear RIPK3 correlated significantly with nuclear pMLKL (Spearman rho: 0.158, *p* = 0.025) and cytoplasmic pMLKL (Spearman rho: 0.271, *p* < 0.001). Nuclear pMLKL correlated significantly with cytoplasmic pMLKL (Spearman rho: 0.363, *p* < 0.001).

In cervical cancer tissue, nuclear p53 not only correlated significantly with nuclear RIPK1 (Spearman rho: 0.274, *p* < 0.001), cytoplasmic RIPK1 (Spearman rho: 0.318, *p* < 0.001) and nuclear RIPK3 (Spearman rho: 0.205, *p* = 0.002) but also with cytoplasmic pMLKL (Spearman rho: 0.171, *p* = 0.014).

hr-HPV protein E7 not only interferes with pRb (retinoblastoma protein) but also with p21, an important regulator of the cell cycle (40). In our study, p21 correlated significantly with proteins relevant for necroptosis: p21 showed a positive correlation with nuclear RIPK1 (Spearman rho: 0.217, *p* = 0.003), cytoplasmic RIPK1 (Spearman rho: 0.311, *p* < 0.001), nuclear RIPK3 (Spearman rho: 0.154, *p* = 0.039) and nuclear pMLKL (Spearman rho: 0.197, *p* = 0.012).

E6 is an important human papilloma virus oncoprotein as one of its key functions is degradation of p53 (18). E6 correlated negatively with nuclear (Spearman rho: *-0.151*, *p* = 0.030) and cytoplasmic (Spearman rho: *-0.157*, *p* = 0.025) pMLKL.

### 3.2 Correlation of RIPK1, RIPK3, and pMLKL with OS and PFS in cervical cancer patients

#### 3.2.1 Nuclear RIPK1 expression correlated with longer OS and PFS in cervical cancer patients

**Figure 3:**
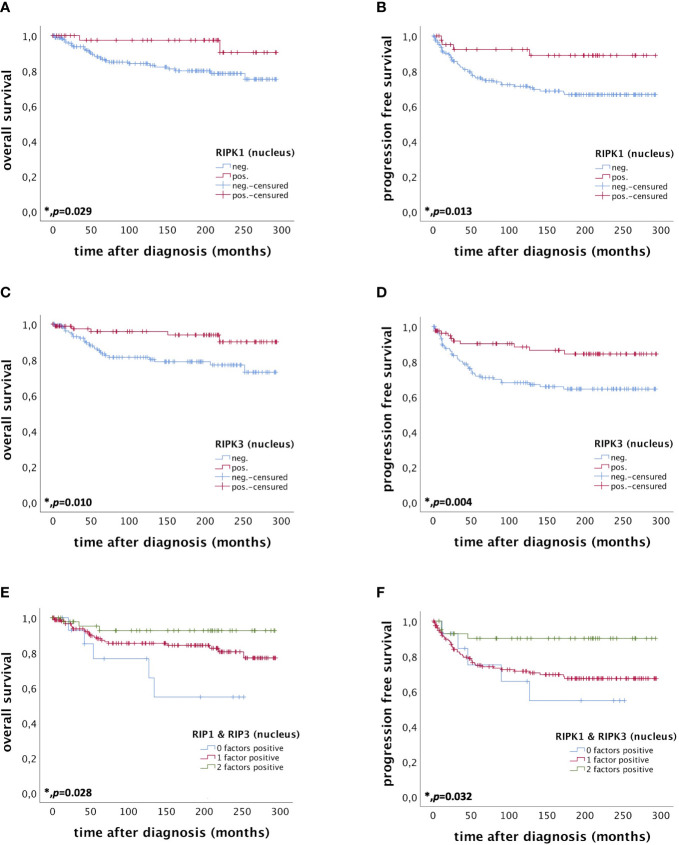
OS and PFS of patients diagnosed with cervical cancer correlated with nuclear RIPK1 expression **(A, B)** nuclear RIPK3 expression **(C, D)** and simultaneous expression of RIPK1 and RIPK3 in the nucleus **(E, F)**. **(A)** OS of patients diagnosed with cervical cancer correlated with nuclear RIPK1 expression. Nuclear RIPK1 expression (n = 47) was associated with longer OS in cervical cancer patients compared to patients not expressing RIPK1 (n = 180) (p = 0.029). **(B)** PFS of patients diagnosed with cervical cancer correlated with nuclear RIPK1 expression. Nuclear RIPK1 expression (n = 43) was associated with longer PFS in cervical cancer patients compared to patients not expressing RIPK1 (n = 172) (p = 0.013). **(C)** OS of patients diagnosed with cervical cancer correlated with nuclear RIPK3 expression. High nuclear RIPK3 expression (n = 86) was associated with longer OS in cervical cancer patients compared to patients with low RIPK3 expression (n = 141) (p = 0.010). **(D)** PFS of patients diagnosed with cervical cancer correlated with nuclear RIPK3 expression. High nuclear RIPK3 expression (n = 81) was associated with longer PFS in cervical cancer patients compared to patients with low RIPK3 expression (n = 134) (p = 0.004). **(E)** OS of patients diagnosed with cervical cancer correlated with simultaneous expression of nuclear RIPK1 and RIPK3. Expression of both RIPK1 and RIPK3 (n = 48) in the nucleus was associated with longer OS in cervical cancer patients (p = 0.028) compared to patients expressing only one (n = 162) or no factor (n = 15). **(F)** PFS of patients diagnosed with cervical cancer correlated with simultaneous expression of nuclear RIPK1 and RIPK3. Expression of both RIPK1 and RIPK3 (n = 44) in the nucleus was associated with longer OS in cervical cancer patients (p = 0.032) compared to patients expressing only one (n = 155) or no factor (n = 14).

Nuclear RIPK1 expression (IRS > 0) was associated with increased survival. Patients expressing RIPK1 in the nucleus had a significantly longer OS (*p* = 0.029) and PFS (*p* = 0.013) (**Figures 3A, B**). Multivariate Cox regression analysis was conducted to test for independent prognosticators for OS and PFS in the cohort of *n* = 250 cervical cancer patients and significantly showed that nuclear RIPK1 expression is an independent prognosticator for OS (**Table 2**) and PFS (**Table 3**).

**Table 2:** Multivariate Cox regression analysis of cervical cancer patients (*n* = 250) and their clinical and pathological characteristics including nuclear RIPK1 expression regarding OS.

covariate	hazard ratio	95% CI	p-value
patient’s age (< 49 yrs vs. ≥ 49 yrs)	2.490	1.173-5.289	0.018*
histological subtype	1.923	1.230-3.005	0.004*
tumor grading	0.977	0.873-1.093	0.683
FIGO	1.022	1.001-1.044	0.040*
nodal status (pNX/0 vs. pN1)	1.514	0.729-3.141	0.266
positive nuclear RIPK1 expression	0.212	0.050-0.893	0.035*

Significant independent factors for OS are indicated with asterisks. (*p < 0.05).

**Table 3:** Multivariate Cox regression analysis of cervical cancer patients (*n* = 250) and their clinical and pathological characteristics including nuclear RIPK1 expression regarding PFS.

covariate	hazard ratio	95% CI	p-value
patient’s age (< 49 yrs vs. ≥ 49 yrs)	1.418	0.810-2.483	0.222
histological subtype	1.647	1.130-2.401	0.009*
tumor grading	0.983	0.902-1.070	0.690
FIGO	1.048	1.024-1.072	< 0.001**
nodal status (pNX/0 vs. pN1)	1.854	1.061-3.240	0.030*
positive nuclear RIPK1 expression	0.304	0.109-0.849	0.023*

Significant independent factors for PFS are indicated with asterisks. (*p < 0.05).

To validate the overall survival and progression-free survival of the RIPK1 gene in a large independent cervical squamous cell carcinoma cohort, the *KM plotter* database was used (41). Sources of the *KM plotter* database include GEO, EGA and TCGA (41). Specifically, the use of GEO and TCGA datasets have already been published on cervical cancer (42, 43). We divided patients into high and low groups of expression of RIPK1 using auto select best cut-off. OS was the chosen to compare these groups. A follow-up threshold of 60 months was chosen. The results showed that the overall survival time of patients in the high-RIPK1 expression group (*n* = 189) was significantly longer than that of the low-expression group (*n* = 115) (*p* = 0.04) (**Figure S3**). The progression-free survival of patients in the high-RIPK1 expression group (*n* = 131) was insignificantly worse compared to patients in the low-expression group (*n* = 43). This correlates with the results of our cohort even though we did not use the C-index, which is a useful tool for analysing different datasets (42, 43).

#### 3.2.2 High nuclear RIPK3 expression correlated with longer OS and PFS in cervical cancer patients

High nuclear RIPK3 expression (IRS > 3) was correlated significantly with a longer OS (*p* = 0.010) as well as a longer PFS (*p* = 0.004) (**Figures 3C, D**). To test for independent prognosticators for OS and PFS in the cohort of *n* = 250 cervical cancer patients multivariate Cox regression analysis was performed. It significantly showed that nuclear RIPK3 expression was an independent prognosticator for PFS (**Table 4**) but not for OS (**Table 5**).

**Table 4:** Multivariate Cox regression analysis of cervical cancer patients (*n* = 250) and their clinical and pathological characteristics including nuclear RIPK3 expression regarding PFS.

covariate	hazard ratio	95% CI	p-value
patient’s age (< 49 yrs vs. ≥ 49 yrs)	1.154	0.654-2.036	0.622
histological subtype	1.408	0.968-2.047	0.073
tumor grading	0.977	0.903-1.057	0.561
FIGO	1.046	1.022-1.070	< 0.001**
nodal status (pNX/0 vs. pN1)	1.966	1.121-3.449	0.018*
positive nuclear RIPK3 expression	0.466	0.288-0.950	0.036*

Significant independent factors for PFS are indicated with asterisks. (*p < 0.05, **p < 0.001).

**Table 5:** Multivariate Cox regression analysis of cervical cancer patients (*n* = 250) and their clinical and pathological characteristics including nuclear RIPK3 expression regarding OS.

covariate	hazard ratio	95% CI	p-value
patient’s age (< 49 yrs vs. ≥ 49 yrs)	1.955	0.915-4.180	0.084
histological subtype	1.596	1.018-2.504	0.042*
tumor grading	0.976	0.885-1.076	0.626
FIGO	1.021	0.999-1.043	0.061
nodal status (pNX/0 vs. pN1)	1.651	0.795-3.427	0.179
positive nuclear RIPK3 expression	0.393	0.147-1.050	0.063

Significant independent factors for OS are indicated with asterisks. (*p < 0.05).

#### 3.2.3 Co-expression of nuclear RIPK1 and RIPK3 correlated with longer OS and PFS in cervical cancer patients

Patients expressing both RIPK1 and RIPK3 in the nucleus (IRS > 0) had a significantly longer OS compared to patients only expressing one or none of these markers (*p* = 0.028). Patients expressing either RIPK1 or RIPK3 had a longer OS than patients without any expression (**Figure 3E**).

Co-expression of RIPK1 and RIPK3 in the nucleus correlated significantly with a longer PFS compared to the expression of only one or none of these two parameters (*p* = 0.032) (**Figure 3F**). Multivariate Cox regression analysis was conducted to test for independent prognosticators for OS and PFS in the cohort of *n* = 250 cervical cancer patients and significantly showed nuclear co-expression of RIPK1 and RIPK3 as independent prognosticators for OS (**Table 6**) and PFS (**Table 7**).

**Table 6:** Multivariate Cox regression analysis of cervical cancer patients (*n* = 250) and their clinical and pathological characteristics including nuclear RIPK1-RIPK3 co-expression regarding OS.

covariate	hazard ratio	95% CI	p-value
patient’s age (< 49 yrs vs. ≥ 49 yrs)	2.336	1.098-4.967	0.028*
histological subtype	1.722	1.101-2.692	0.017*
tumor grading	0.981	0.882-1.090	0.715
FIGO	1.024	1.003-1.046	0.028*
nodal status (pNX/0 vs. pN1)	1.655	0.803-3.415	0.172
positive nuclear co- expression of RIPK1 and RIPK3	0.386	0.187-0.797	0.010*

Significant independent factors for OS are indicated with asterisks. (*p < 0.05).

**Table 7:** Multivariate Cox regression analysis of cervical cancer patients (*n* = 250) and their clinical and pathological characteristics including nuclear RIPK1-RIPK3 co-expression regarding PFS.

covariate	hazard ratio	95% CI	p-value
patient’s age (< 49 yrs vs. ≥ 49 yrs)	1.369	0.779-2.406	0.274
histological subtype	1.484	1.020-2.161	0.039*
tumor grading	0.985	0.906-1.071	0.719
FIGO	1.048	1.025-1.073	< 0.001**
nodal status (pNX/0 vs. pN1)	1.945	1.111-3.408	0.020*
positive nuclear co- expression of RIPK1 and RIPK3	0.521	0.296-0.917	0.024*

Significant independent factors for PFS are indicated with asterisks. (*p < 0.05, **p < 0.001).

#### 3.2.4 Co-expression of RIPK1 and RIPK3 and pMLKL is a positive prognosticator for OS in cervical cancer patients

**Figure 4:**
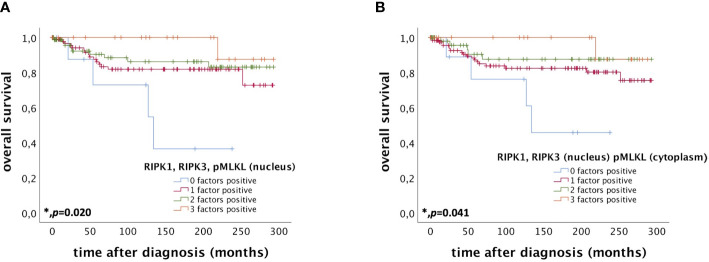
OS of patients diagnosed with cervical cancer correlated with co-expression of RIPK1, RIPK3, and pMLKL. **(A)** OS of patients diagnosed with cervical cancer correlated with co-expression of RIPK1, RIPK3, and pMLKL in the nucleus. Expression of all three factors (RIPK1, RIPK3, and pMLKL) (n = 22) in the nucleus was associated with longer OS in cervical cancer patients (p = 0.020) compared to patients expressing only two (n = 75), one (n = 97) or no factor (n = 9). **(B)** OS of patients diagnosed with cervical cancer correlated with co-expression of nuclear RIPK1 and RIPK3 and cytoplasmic pMLKL. Expression of all three factors (nuclear RIPK1, nuclear RIPK3 and cytoplasmic pMLKL) (n = 20) was associated with longer OS in cervical cancer patients (p = 0.041) compared to patients expressing only two (n = 51), one (n = 122) or no factor (n = 10).

Patients expressing RIPK1, RIPK3, and pMLKL in the nucleus (IRS > 0) had a significantly longer OS compared to patients only expressing two, one or none of these factors (*p* = 0.020) (**Figure 4A**). The more factors a cervical cancer patient of this cohort expressed, the longer the OS.

Co-expression of RIPK1 and RIPK3 in the nucleus and cytoplasmic pMLKL (IRS > 0) also correlated with a longer OS compared to patients only expressing two, one or none of these factors (*p* = 0.041) (**Figure 4B**).

We performed multivariate Cox regression analysis to test for independent prognosticators for OS and PFS in the cohort of *n* = 250 cervical cancer patients. It significantly showed nuclear co-expression of RIPK1, RIPK3, and pMLKL as an independent prognosticator for OS (**Table 8**). In another multivariate Cox regression analysis, co-expression of nuclear RIPK1, nuclear RIPK3 and cytoplasmic pMLKL presented as a significant independent prognosticator for OS in cervical cancer patients (**Table 9**).

**Table 8:** Multivariate Cox regression analysis of cervical cancer patients (*n* = 250) and their clinical and pathological characteristics including nuclear RIPK1-RIPK3-pMLKL co-expression regarding OS.

covariate	hazard ratio	95% CI	p-value
patient’s age (< 49 yrs vs. ≥ 49 yrs)	1.995	0.911-4.368	0.084
histological subtype	1.849	1.188-2.878	0.006*
tumor grading	0.980	0.881-1.089	0.704
FIGO	1.022	1.000-1.044	0.052
nodal status (pNX/0 vs. pN1)	2.071	0.974-4.405	0.059
positive nuclear RIPK1, RIPK3, and pMLKL expression	0.573	0.340-0.964	0.036*

Significant independent factors for OS are indicated with asterisks. (*p < 0.05).

**Table 9:** Multivariate Cox regression analysis of cervical cancer patients (*n* = 250) and their clinical and pathological characteristics including nuclear RIPK1-RIPK3 and cytoplasmic pMLKL co-expression regarding OS.

covariate	hazard ratio	95% CI	p-value
patient’s age (< 49 yrs vs. ≥ 49 yrs)	2.337	1.064-5.131	0.034*
histological subtype	1.959	1.258-3.051	0.003*
tumor grading	0.979	0.882-1.088	0.694
FIGO	1.023	1.001-1.045	0.041*
nodal status (pNX/0 vs. pN1)	1.770	0.823-3.807	0.144
positive nuclear RIPK1 and RIPK3 and cytoplasmic pMLKL expression	0.492	0.272-0.890	0.019*

Significant independent factors for OS are indicated with asterisks. (*p < 0.05).

#### 3.2.5 Nuclear pMLKL expression is a positive prognosticator for PFS in the subgroup of grading G2 cervical cancer patients

In the subgroup of grading G2 cervical cancer, patients expressing nuclear pMLKL (IRS > 1) had a tendentially longer OS (p = 0.056) (as shown in Figure S1A). Furthermore, patients diagnosed with grading G2 cervical cancer expressing nuclear pMLKL (IRS > 0) had a significantly longer PFS (p = 0.043) (as shown in **Figure S1B**).

### 3.3 Cell viability and cell proliferation

#### 3.3.1 C2 ceramide reduces cell viability and cell proliferation

In a previous study we could show that C2 ceramide can induce necroptosis in human luteal granulosa cells (36). To investigate C2 ceramide’s effect on viability and cell proliferation in cervical cancer cells we conducted MTT and BrdU assays.

**Figure 5:**
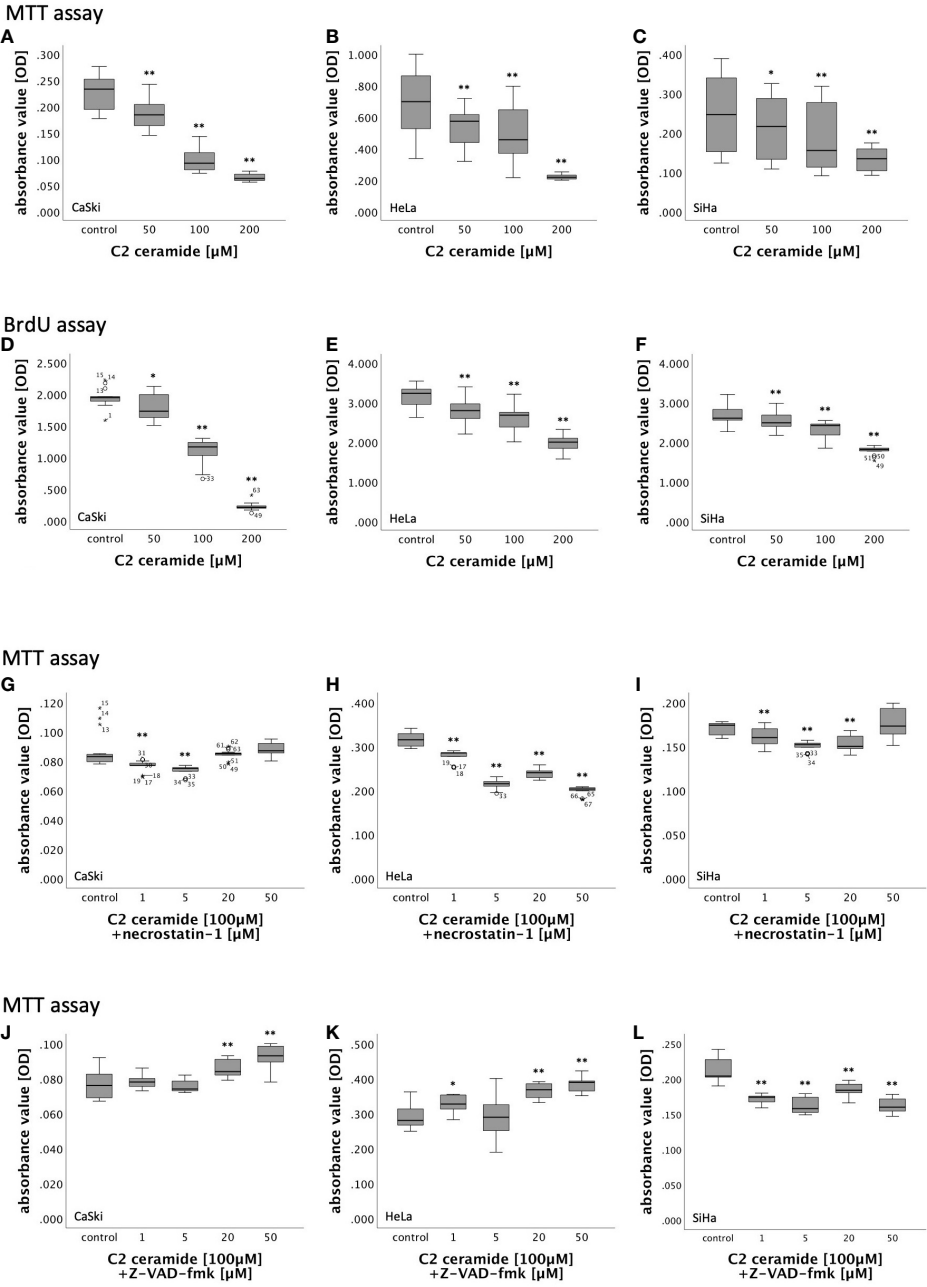
**(A–F)** MTT- and BrdU assay results of cervical cancer cell lines (CaSki, HeLa, SiHa) after 72h stimulation with C2 ceramide (50µM, 100µM, 200µM) compared to the respective DMSO control. **(G–L)** MTT assay results of cervical cancer cell lines (CaSki, HeLa, SiHa) after 48h stimulation with C2 ceramide (100µM) and necrostatin-1 (1µM, 5µM, 20µM and 50µM) compared to the respective DMSO control **(G–I)** and after 72h stimulation with C2 ceramide (100µM) and Z-VAD-fmk (1µM, 5µM, 20µM and 50µM) compared to the respective DMSO control **(J–L)** (for each concentration respectively control: n = 15, **p < 0.001, *p < 0.05).

In MTT assay, CaSki, HeLa and SiHa cells showed a concentration-dependent decreasing viability after stimulation with 50µM, 100µM and 200µM C2 ceramide after 24h, 48h and 72h in comparison to the corresponding DMSO control (**Figures 5A–C**; **Figure S3**).

BrdU assay was performed for 48h and 72h because the effect of C2 ceramide in MTT assay was most present in these time frames. While stimulation of CaSki cells with C2 ceramide led to a significantly concentration-dependent decrease of cell proliferation after both 48h and 72h, cell proliferation in HeLa and SiHa cells was concentration-dependently inhibited only after 72h (**Figures 5D–F**; **Figure S4**).

#### 3.3.2 Necrostatin-1 did not significantly inhibit the cell viability reductive effect of C2 ceramide

Nec-1 is an inhibitor of RIPK1. To identify the form of induced cell death caused by C2 ceramide, simultaneous stimulation of C2 ceramide and nec-1 or C2 ceramide and the pan-caspase inhibitor Z-VAD-fmk, respectively, was conducted. For that, CaSki, HeLa and SiHa cells were incubated with different concentrations of nec-1 (1µM, 5µM, 20µM and 50µM) followed by stimulation with 100µM C2 ceramide.

In HeLa cells, incubation with nec-1 did not lead to inhibition of the reduction of cell viability caused by C2 ceramide. Instead, cell viability decreased with higher concentration of nec-1. After 48h, co-stimulation of 100µM C2 ceramide and 50µM nec-1 showed a slight increase in CaSki and SiHa cell viability compared to the solvent control, which was insignificant. All other concentrations in all other time frames showed a decrease in cell viability (**Figures 5G–I**; **Figure S5**).

#### 3.3.3 Z-VAD-fmk partially inhibited C2 ceramide’s reductive effect on cell viability

CaSki, HeLa and SiHa cells were incubated with different concentrations of Z-VAD-fmk (1µM, 5µM, 20µM and 50µM) followed by stimulation with 100µM C2 ceramide.

In both CaSki and HeLa cells, co-stimulation of Z-VAD-fmk and C2 ceramide led to a significant concentration-dependent increase in viability after 72h. Co-stimulation of Z-VAD-fmk and C2 ceramide decreased cell viability in SiHa cells (**Figures 5J–L**).

### 3.4 Live-cell imaging showed necroptotic morphological changes in CaSki and HeLa cell lines stimulated with C2 ceramide

Live-cell imaging was performed over 72h after stimulation with C2 ceramide to investigate morphological changes. Incubation of CaSki and HeLa cells with C2 ceramide showed a visible reduction of confluency, and ballooning of the cells was observed. Treatment of SiHa cells with C2 ceramide did not have any visible effect on the morphology of cervical cancer cells.

### 3.5 C2 ceramide induced apoptosis and necroptosis in AnnexinV-FITC apoptosis staining followed by FACS analysis

**Figure 6:**
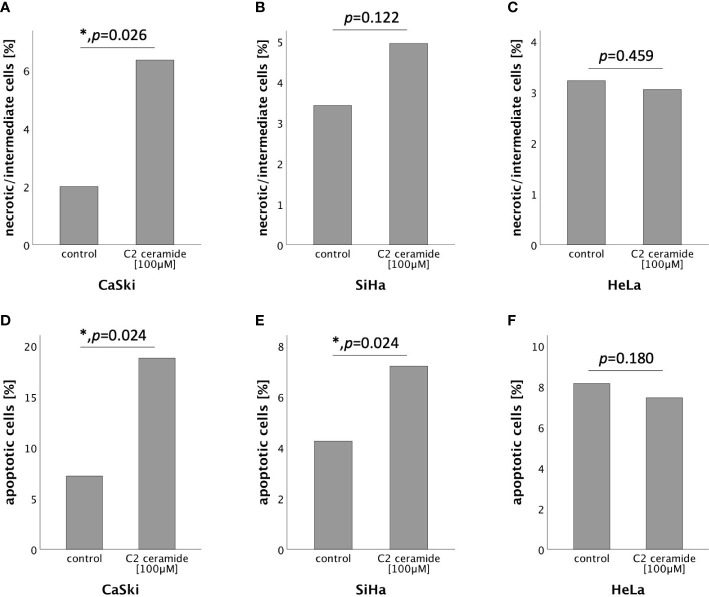
AnnexinV-FITC apoptosis staining followed by FACS comparing the percentual number of necrotic/intermediate cells of the control with the C2 ceramide treated cells **(A–C)**. AnnexinV-FITC apoptosis staining followed by FACS comparing the percentual number of apoptotic cells of the control with the C2 ceramide treated cells **(D–F)**.

To further specify the type of cell death induced by C2 ceramide, AnnexinV-FITC apoptosis staining followed by FACS analysis was performed after 72h stimulation with C2 ceramide (100μM). A significant percentual increase in apoptotic as well as in necrotic/intermediate (dying) cells compared to the respective control was observed in CaSki cells (**Figures 6A, D**). In SiHa cells, a significant increase in apoptotic but not in necrotic/intermediate (dying) cells was observed (**Figures 6B, E**). Treatment of HeLa cells with C2 ceramide did not show a significant percentual increase in apoptotic or necrotic/intermediate (dying) cells (**Figures 6C, F**).

### 3.6 RIPK3 expression in CaSki, HeLa and SiHa cells

The results of Western Blot showed that RIPK3 of expected size (~57kDa) was detected in all three cell lines examined (**Figure S8**).

## 4 Discussion

Necroptosis has been of growing interest in recent years. It was long considered as a “back-up” cell death mechanism in the TNFR1 pathway in case casp8 was blocked but since necroptosis can be induced not only *via* TNFR1 but also TNFR1-independently *via* ZBP1 and other receptors, the concept of necroptosis being only a “back-up” is being discussed (32). In the model of a hr-HPV-infected cell, this concept might still be of interest since the oncoprotein E6 binds to TNFR1 (44). Hr-HPV can block apoptosis and necroptosis (20, 45, 46). Inhibition of apoptosis through hr-HPV can either function *via* an increase of Bcl-2 and decrease of BAK caused by E6 and E7 (45) or by binding of the large isoform of E6 to procasp8, accelerating its degradation (46). Inhibition or degradation of casp8 prevents cleavage of RIPK1 and RIPK3 by the procasp8- cFLIP_L_ heterodimer allowing autophosphorylation of RIPK3 and the induction of necroptosis (32). Since infection with hr-HPV also leads to downregulation of RIPK3 expression through a yet unknown mechanism, necroptosis is also blocked (20). The inhibition of necroptosis might enable infected cells to partially evade the effector mechanisms of the immune system and thereby facilitate a persistent infection with hr-HPV and ultimately, the progression of hr-HPV-induced lesions (20). Consistent with this study depicting necroptosis as a positive mechanism in cervical cancer, we found that patients with high nuclear RIPK3 expression, a key factor in the necroptotic pathway, had a significantly longer OS as well as PFS compared to patients with low expression of nuclear RIPK3. High nuclear RIPK3 expression was significantly associated with lower FIGO classification, low extent of the primary tumor (pT1) and negative lymph node status (pN0) in cervical cancer. This is supported by the findings indicating that RIPK3 is active in the nucleus and that nuclear RIPK3 is involved in necroptosis (47). Hence, we conclude that downregulation of RIPK3 *via* hr-HPV has a negative prognostic impact on OS and PFS in cervical cancer patients.

If necroptosis is induced *via* TNFR1, RIPK1 plays a key role in recruiting RIPK3 (21). In cervical cancer patients, expression of nuclear RIPK1 is an independent positive prognosticator for OS and PFS. Simultaneous expression of RIPK1 and RIPK3 in the nucleus is an independent positive prognosticator for OS and PFS further underlining a potential positive effect of necroptosis in cervical cancer patients.

RIPK3 phosphorylates MLKL in the nucleus (47), followed by translocation to intracellular and plasma membranes leading to disruption of membrane integrity and ultimately necroptotic cell death (26, 48, 49). In our study, we could show that expression of nuclear pMLKL is a positive prognosticator for PFS in patients diagnosed with G2 cervical cancer and that expression of cytoplasmic pMLKL is associated with negative regional lymph node involvement (pN0). While the expression of pMLKL seems to positively impact cervical cancer patients, high levels of pMLKL are associated with poor OS in esophageal and colon cancer (50). This discrepancy in OS in different cancer types might be explained by different mechanisms of tumorigenesis with persistent hr-HPV infection being the leading cause of cervical cancer development (18). A reason for worse OS and PFS in cervical cancer patients not expressing the proteins necessary for necroptosis could be that E6 and E7 immortalize infected cells, targeting crucial regulators of proliferation and genomic stability (19), which might lead to the progression of HPV-induced lesions.

The presence of all three factors important for necroptosis (RIPK3, RIPK1, and pMLKL) is associated with a significantly longer OS compared to patients only expressing two, one or none of these factors. Since the expression of all three factors necessary for necroptosis is associated with longer OS, necroptosis could effectively regulate persistent hr-HPV infections and the progression of hr-HPV-induced lesions.

Persistent infection with hr-HPV not only leads to degradation of p53 through viral protein E6 (19) but also resists the induction of necroptosis in keratinocytes (20). p53 is an essential regulator of the cell cycle, acting as a tumor suppressor, and plays an important role in the regulation of necroptosis by leading to an increase of RIPK1 and RIPK3 through activation of necrosis-related factor (NRF) expression in cardiomyocytes (51). Consistent with these studies, we showed that expression of p53 correlated significantly with expression of nuclear and cytoplasmic RIPK1, nuclear RIPK3 and cytoplasmic pMLKL in cervical cancer tissue. Furthermore, E6 correlated negatively with nuclear and cytoplasmic pMLKL. hr-HPV oncoprotein E7 inactivates p21, an important cell cycle regulator and tumor suppressor (40). In this study, expression of p21 significantly correlated with most proteins relevant for necroptosis (nuclear and cytoplasmic RIPK1, nuclear RIPK3, nuclear pMLKL). The correlation of p53 and p21 with several proteins relevant for necroptosis indicates that insufficient degradation or insufficient inactivation of p53 and p21 through hr-HPV is associated with the preserved capability of necroptosis. We could also show strong positive correlation of all three examined factors (RIPK1, RIPK3, pMLKL) with each other. This could indicate that cervical cancer cells are capable of inducing necroptosis, specifically in those where RIPK3 is not downregulated.

Ceramides are biologically active sphingolipid metabolites that are important for cell death regulation in cancer cells, and the potential of ceramide analogs for treatment of ovarian cancer is currently being tested in several dose-escalating phase 1 clinical trials (33, 52). In human head and neck squamous cell carcinoma and human hepatocellular carcinoma, ceramide species are reduced (53, 54). Through induction of programmed cell death, ceramides, such as C2 ceramide, present as a potent tumor suppressor and, therefore, as a possible therapeutic strategy (55–58). In our study, C2 ceramide concentration-dependently reduced viability and proliferation in the cervical cancer cell lines CaSki, HeLa, and SiHa leading to the assumption that cell death can be induced by C2 ceramide in cervical cancer cells. Morphological changes after stimulation with C2 ceramide were evaluated using live-cell imaging. In CaSki and HeLa cells, signs typical for necro(pto)tic cell death, such as ballooning and reduction of confluency, could be detected after stimulation with C2 ceramide, whereas treatment of SiHa cells did not have any visible effects. To further identify the form of induced cell death, we tried to neutralize C2 ceramide’s effect by simultaneous stimulation of C2 ceramide together with the RIPK1 inhibitor nec-1 or the pan-caspase inhibitor Z-VAD-fmk. Nec-1 did not significantly reverse C2 ceramide’s effect on cell viability but showed a tendency (*p*=0.108) in SiHa cells when co-stimulated with 50nM of nec-1 for 48h. This is in favor of a necroptotic event. Z-VAD-fmk significantly inhibited C2 ceramide’s effect in CaSki and HeLa cells after 72h.

Feoktistova et al. (2011) postulated that HeLa cells lack basal RIPK3 expression (59). We could show that HeLa cells, as well as CaSki and SiHa cells, express sufficient levels of RIPK3, potentially enabling CaSki, HeLa and SiHa cells to necroptosis. AnnexinV-FITC apoptosis staining followed by Flow Cytometry proved a significant increase in apoptotic CaSki and SiHa cells after treatment with C2 ceramide compared to the control. It also showed a significant increase in necrotic/intermediate (dying) CaSki cells compared to the control. C2 ceramide induces necroptosis in human luteal granulosa cells (36). It can also induce apoptosis in human malignant glioma cells (58) and human colon carcinoma cells (60). In head and neck squamous cell carcinoma, both necroptosis and apoptosis are induced by C2 ceramide (57). It is possible that C2 ceramide induces cell death independently of RIPK1 and that this is the reason why simultaneous stimulation of C2 ceramide and the RIPK1 inhibitor nec-1 did not show any inhibitory effects of C2 ceramide in the MTT assay. Taken together, these are indicators that C2 ceramide induces both necroptosis and apoptosis in the two cervical cancer cell lines CaSki and HeLa. Further studies are required to identify the exact mechanism by which C2 ceramide induces cell death.

In conclusion, this study showed that RIPK1 and RIPK3 correlated with longer OS and PFS in cervical cancer patients and can therefore be described as positive prognosticators for cervical cancer. pMLKL was associated with longer PFS in the subgroup of G2 grading. These results comply with the current understanding of hr-HPV infection, which can inhibit programmed cell deaths to evade effector mechanisms of the immune system and thereby potentially promote tumorigenesis (20, 45, 46). This study further elucidated the effect of C2 ceramide on cell viability and cell proliferation. Consistent with *in vitro* studies in other cancer cell lines (36, 57, 58, 60), C2 ceramide reduced cell viability and cell proliferation in cervical cancer cell lines. AnnexinV-FITC apoptosis staining followed by FACS analysis supported the results indicating that cell viability is reduced through the induction of both apoptosis and necroptosis.

## Conflicts of interest

The authors declare that the research was conducted in the absence of any commercial or financial relationships that could be construed as a potential conflict of interest.

## Data availability statement

The original contributions presented in the study are included in the article/**Supplementary Material**. Further inquiries can be directed to the corresponding author.

## Ethics statement

The studies involving human participants were reviewed and approved by ethics committee of Ludwig-Maximilians University of Munich. Written informed consent for participation was not required for this study in accordance with the national legislation and the institutional requirements.

## Author contributions

AV, AM and UJ designed and conceived the project, provided the concept, edited the manuscript, and supervised the research. TV performed most experiments, all statistical evaluation and wrote the manuscript. VK and SJ performed experiments and contributed to editing of the manuscript. ES, KB, KE, DM, AT, NT and SM contributed to the editing of the manuscript. All authors contributed to the article and approved the submitted version.
