# Supplementary material for: RIPK1 and RIPK3 are positive prognosticators for cervical cancer patients and C2 ceramide can inhibit tumor cell proliferation in vitro
Source: Front Oncol. 2023 May 1;13:1110939. doi: 10.3389/fonc.2023.1110939 (PMC10183606; doi:10.3389/fonc.2023.1110939)
Supplement: Supplementary file 1 [file DataSheet_1.docx]

Supplementary Material

**Table S1.** Correlation of RIPK1 expression and various histopathological variables.

|  | **RIPK1 (nucleus)** | | **RIPK1 (cytoplasm)** | |
| --- | --- | --- | --- | --- |
|  | **Correlation coefficient** | ***p*** | **Correlation coefficient** | ***p*** |
| RIPK1 (nucleus) | - | - | 0.486 | **< 0.001 **** |
| RIPK1 (cytoplasm) | 0.486 | **< 0.001 **** | - | - |
| RIPK3 (nucleus) | 0.414 | **< 0.001 **** | 0.471 | **< 0.001 **** |
| pMLKL (nucleus) | 0.184 | **0.009 *** | 0.168 | **0.016 *** |
| pMLKL (cytoplasm) | 0.310 | **< 0.001 **** | 0.278 | **< 0.001 **** |
| E6 (cytoplasm) | *-0.040* | 0.554 | *-0.045* | 0.506 |
| p53 (nucleus) | 0.274 | **< 0.001 **** | 0.318 | **< 0.001 **** |
| p53 (cytoplasm) | 0.131 | 0.052 | 0.035 | 0.607 |
| p21 | 0.217 | **0.003 *** | 0.311 | **< 0.001 **** |

**Table S2.** Correlation of nuclear RIPK3 expression and various histopathological variables.

|  | **Correlation coefficient** | ***p*** |
| --- | --- | --- |
| RIPK1 (nucleus) | 0.414 | **< 0.001 **** |
| RIPK1 (cytoplasm) | 0.471 | **< 0.001 **** |
| RIPK3 (nucleus) | - | - |
| pMLKL (nucleus) | 0.158 | **0.025 *** |
| pMLKL (cytoplasm) | 0.271 | **< 0.001 **** |
| E6 (cytoplasm) | 0.042 | 0.534 |
| p53 (nucleus) | 0.205 | **0.002 *** |
| p53 (cytoplasm) | *-0.028* | 0.674 |
| p21 | 0.154 | **0.039 *** |

**Table S3.** Correlation of pMLKL expression and various histopathological variables.

|  | **pMLKL (nucleus)** | | **pMLKL (cytoplasm)** | | |
| --- | --- | --- | --- | --- | --- |
|  | **Correlation coefficient** | ***p*** | **Correlation coefficient** | ***p*** |  |
| RIPK1 (nucleus) | 0.184 | **0.009 *** | 0.310 | **< 0.001 **** |  |
| RIPK1 (cytoplasm) | 0.168 | **0.016 *** | 0.278 | **< 0.001 **** |  |
| RIPK3 (nucleus) | 0.158 | **0.025 *** | 0.271 | **< 0.001 **** |  |
| pMLKL (nucleus) | - | - | 0.363 | **< 0.001 **** |  |
| pMLKL (cytoplasm) | 0.363 | **< 0.001 **** | - | - |  |
| E6 (cytoplasm) | *-0.151* | **0.030 *** | *-0.157* | **0.025 *** |  |
| p53 (nucleus) | 0.024 | 0.732 | 0.171 | **0.014 *** |  |
| p53 (cytoplasm) | 0.096 | 0.169 | 0.216 | **0.002 *** |  |
| p21 | 0.197 | **0.012 *** | 0.121 | 0.119 |  |

**Figure S1.** OS and PFS of patients diagnosed with cervical cancer in the subgroup grading G2 correlated with nuclear pMLKL. **(a)** OS of patients diagnosed with cervical cancer grading G2 correlated with nuclear pMLKL expression. High nuclear pMLKL (IRS > 1) expression (n = 18) was tendentially associated with better OS in cervical cancer grading G2 patients compared to patients not expressing pMLKL (n = 102) (p = 0.056). **(b)** PFS of patients diagnosed with cervical cancer grading G2 correlated with nuclear pMLKL expression. Nuclear pMLKL (IRS > 0) expression (n = 37) was significantly associated with better PFS in cervical cancer patients compared to patients not expressing pMLKL (n = 80) (p = 0.043).

**Figure S2.** MTT assay results of cervical cancer cell lines (CaSki, HeLa, SiHa) displaying a gradual reduction of cell viability after stimulation with C2 ceramide (50µM, 100µM, 200µM) compared to the respective DMSO control (for each concentration respectively control: n = 15), ** p < 0.001, * p < 0.05 (A-C) CaSki (A), HeLa (B) and SiHa (C) cells stimulated with C2 ceramide (50µM, 100µM, 200µM) for 24h in comparison to the respective DMSO control. (D-F) CaSki (D), HeLa (E) and SiHa (F) cells stimulated with C2 ceramide (50µM, 100µM, 200µM) for 48h in comparison to the respective DMSO control.

**Figure S3.** BrdU assay results of cervical cancer cell lines (CaSki, HeLa, SiHa) after stimulation with C2 ceramide (50µM, 100µM, 200µM) compared to the respective DMSO control (for each concentration respectively control: n = 15), ** p < 0.001, * p < 0.05 (A-C) CaSki (A), HeLa (B) and SiHa (C) cells stimulated with C2 ceramide (50µM, 100µM, 200µM) for 48h in comparison to the respective DMSO control.

**Figure S4.** MTT assay results of cervical cancer cell lines (CaSki, HeLa, SiHa) after stimulation with C2 ceramide (100µM) and necrostatin-1 (1µM, 5µM, 20µM and 50µM) compared to the respective DMSO control (for each concentration respectively control: n = 15), ** p < 0.001, * p < 0.05 (A-C) CaSki (A), HeLa (B) and SiHa (C) cells stimulated with C2 ceramide (100µM) and nec-1 (1µM, 5µM, 20µM and 50µM) for 24h in comparison to the respective DMSO control. (D-F) CaSki (D), HeLa (E) and SiHa (F) cells stimulated with C2 ceramide (100µM) and nec-1 (1µM, 5µM, 20µM and 50µM) for 72h in comparison to the respective DMSO control.

**Figure S5.** MTT assay results of cervical cancer cell lines (CaSki, HeLa, SiHa) after stimulation with C2 ceramide (100µM) and Z-VAD-fmk (1µM, 5µM, 20µM and 50µM) compared to the respective DMSO control (for each concentration respectively control: n = 15), ** p < 0.001, * p < 0.05 (A-C) CaSki (A), HeLa (B) and SiHa (C) cells stimulated with C2 ceramide (100µM) and Z-VAD-fmk (1µM, 5µM, 20µM and 50µM) for 24h in comparison to the respective DMSO control. (D-F) CaSki (D), HeLa (E) and SiHa (F) cells stimulated with C2 ceramide (100µM) and Z-VAD-fmk (1µM, 5µM, 20µM and 50µM) for 48h in comparison to the respective DMSO control.
