# Supplementary material for: RIPK1 and RIPK3 are positive prognosticators for cervical cancer patients and C2 ceramide can inhibit tumor cell proliferation in vitro
Source: Front Oncol. 2023 May 1;13:1110939. doi: 10.3389/fonc.2023.1110939 (PMC10183606; doi:10.3389/fonc.2023.1110939)
Supplement: Supplementary file 2 [file Presentation_1.pptx]

## Slide 1
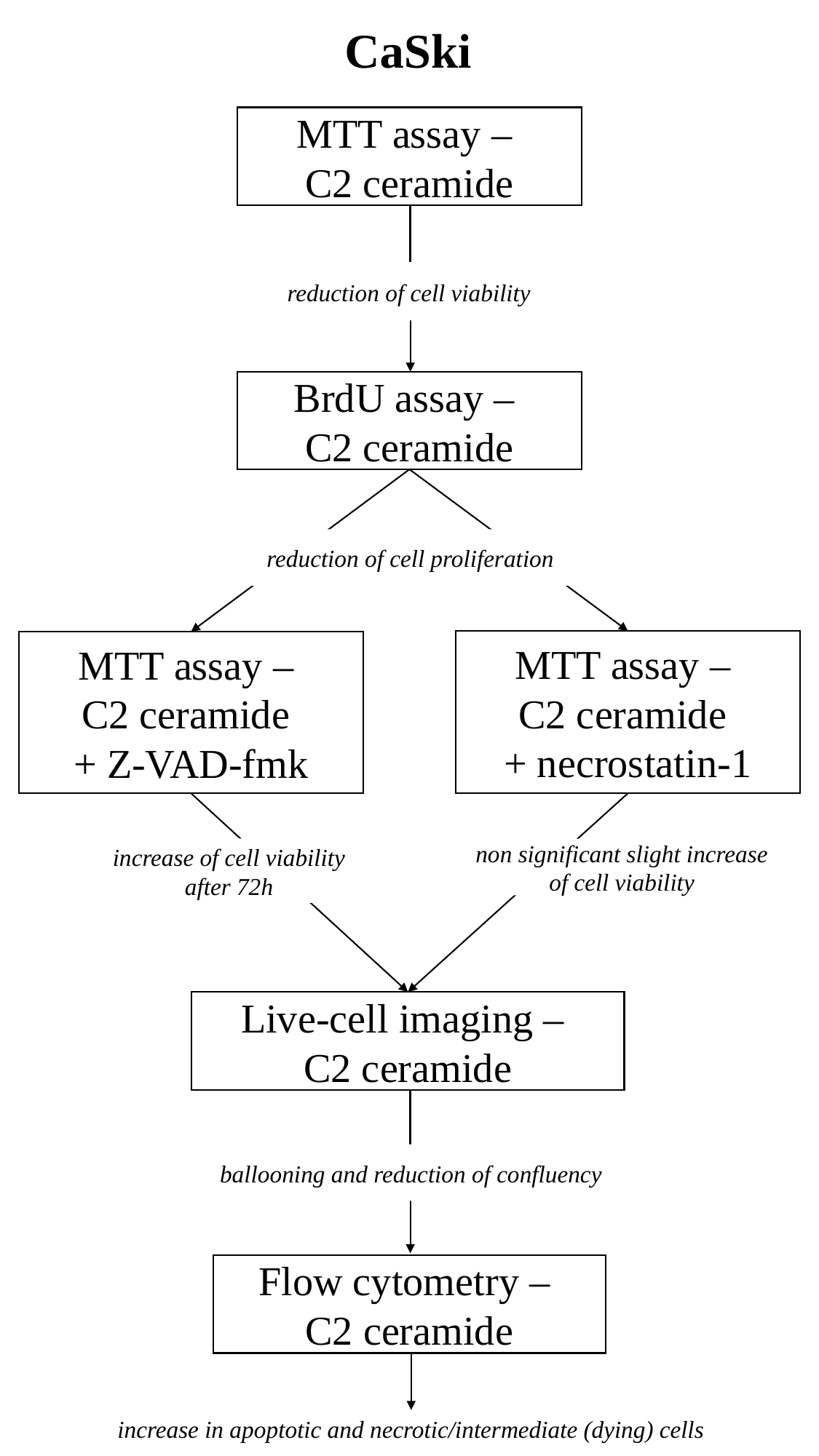

CaSki
MTT assay –
C2 ceramide
reduction of cell viability
BrdU assay –
C2 ceramide
reduction of cell proliferation
MTT assay –
C2 ceramide
+ necrostatin-1
MTT assay –
C2 ceramide
+ Z-VAD-fmk
increase of cell viability after 72h
non significant slight increase of cell viability
Live-cell imaging –
C2 ceramide
ballooning and reduction of confluency
Flow cytometry –
C2 ceramide
increase in apoptotic and necrotic/intermediate (dying) cells

## Slide 2
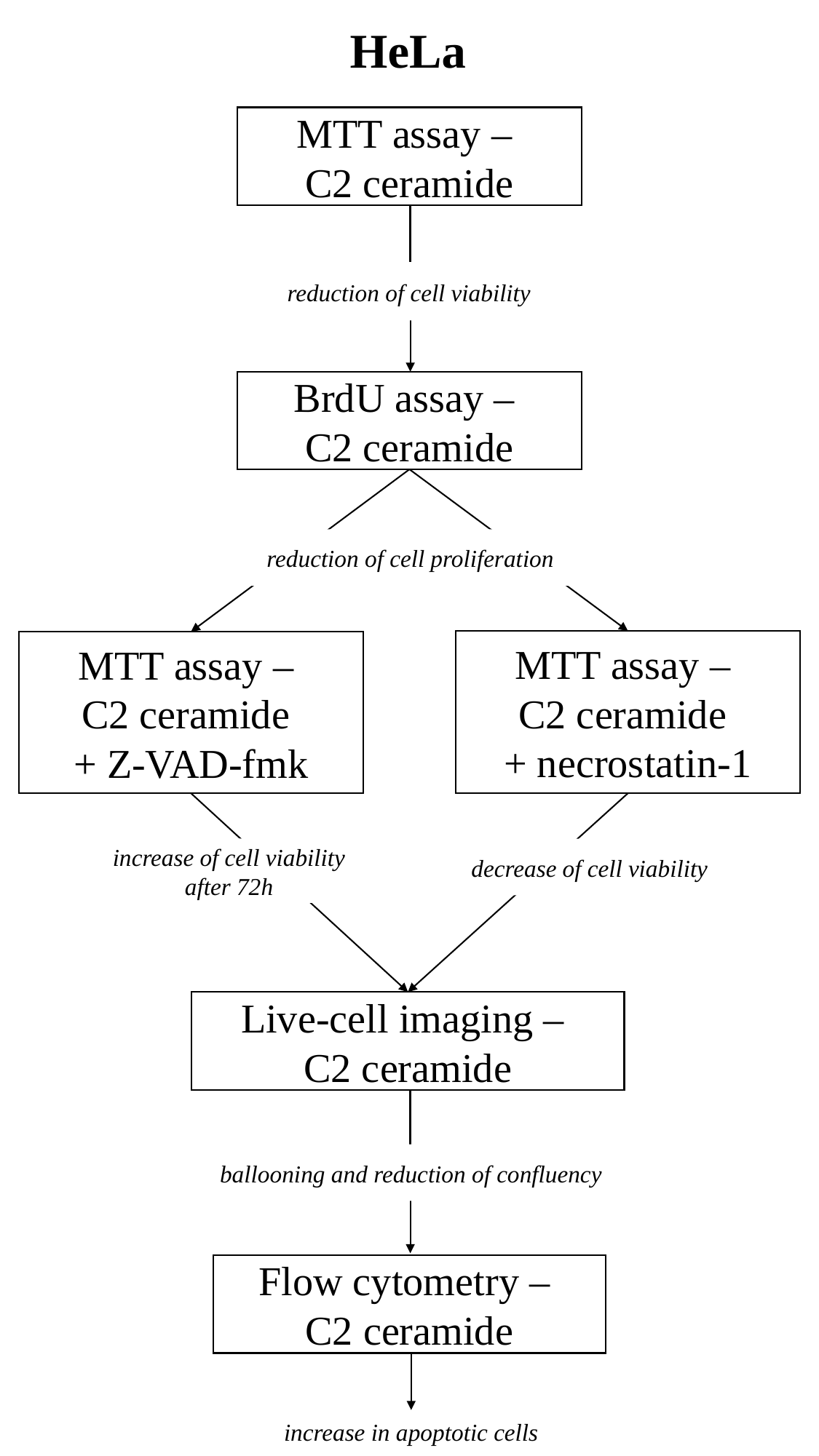

HeLa
MTT assay –
C2 ceramide
reduction of cell viability
BrdU assay –
C2 ceramide
reduction of cell proliferation
MTT assay –
C2 ceramide
+ necrostatin-1
MTT assay –
C2 ceramide
+ Z-VAD-fmk
increase of cell viability after 72h
decrease of cell viability
Live-cell imaging –
C2 ceramide
ballooning and reduction of confluency
Flow cytometry –
C2 ceramide
increase in apoptotic cells

## Slide 3
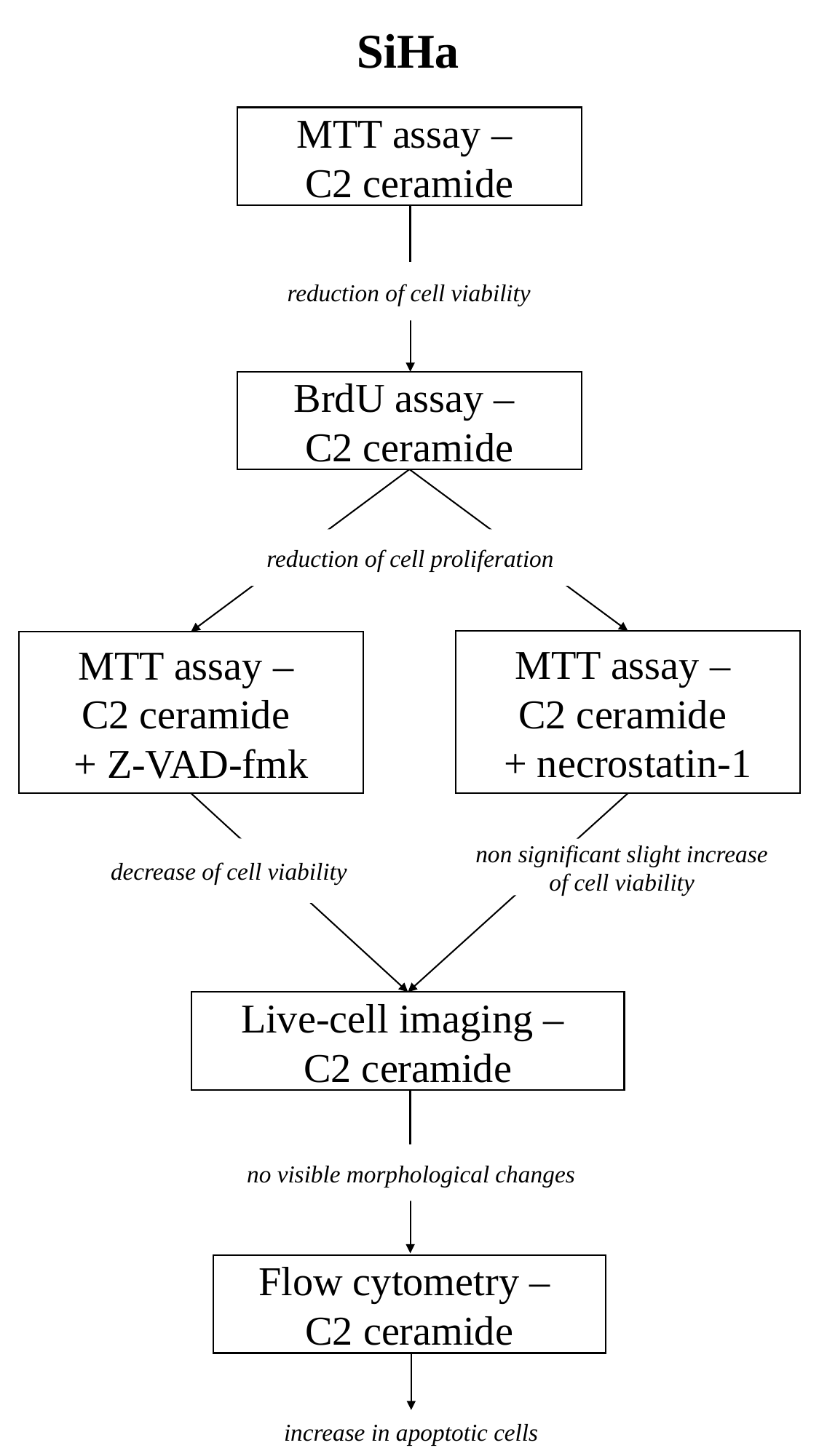

SiHa
MTT assay –
C2 ceramide
reduction of cell viability
BrdU assay –
C2 ceramide
reduction of cell proliferation
MTT assay –
C2 ceramide
+ necrostatin-1
MTT assay –
C2 ceramide
+ Z-VAD-fmk
decrease of cell viability
non significant slight increase of cell viability
Live-cell imaging –
C2 ceramide
no visible morphological changes
Flow cytometry –
C2 ceramide
increase in apoptotic cells
